# SPECT/CT imaging of the lumbar spine in chronic low back pain: a case report

**DOI:** 10.1186/2045-709X-19-2

**Published:** 2011-01-11

**Authors:** Michael H Carstensen, Mashael Al-Harbi, Jean-Luc Urbain, Tarik-Zine Belhocine

**Affiliations:** 1 Department of Medical Imaging, St Joseph's Hospital London, Ontario, Canada

## Abstract

Mechanical low back pain is a common indication for Nuclear Medicine imaging. Whole-body bone scan is a very sensitive but poorly specific study for the detection of metabolic bone abnormalities. The accurate localisation of metabolically active bone disease is often difficult in 2D imaging but single photon emission computed tomography/computed tomography (SPECT/CT) allows accurate diagnosis and anatomic localisation of osteoblastic and osteolytic lesions in 3D imaging. We present a clinical case of a patient referred for evaluation of chronic lower back pain with no history of trauma, spinal surgery, or cancer. Planar whole-body scan showed heterogeneous tracer uptake in the lumbar spine with intense localisation to the right lateral aspect of L3. Integrated SPECT/CT of the lumbar spine detected active bone metabolism in the right L3/L4 facet joint in the presence of minimal signs of degenerative osteoarthrosis on CT images, while a segment demonstrating more gross degenerative changes was more quiescent with only mild tracer uptake. The usefulness of integrated SPECT/CT for anatomical and functional assessment of back pain opens promising opportunities both for multi-disciplinary clinical assessment and treatment for manual therapists and for research into the effectiveness of manual therapies.

## Background

The concept of lumbar facet joints causing or contributing to mechanical low back pain syndromes has been debated in the health care literature for decades [1]. Practitioners of the various manual therapies commonly treat patients presenting with low back pain but are faced with the diagnostic challenge of trying to identify a tissue source of low back pain. While this complaint may be the result of any of a number of pathologies, the vast majority of low back pain falls under the diagnostic umbrella of '' mechanical low back pain '' [2]. We present here the case of a patient with radiological signs of marked lumbosacral junction facet joint osteoarthrosis and clinical symptoms supportive of pathology in this region but with SPECT/CT findings suggestive of an active bony lesion at a more remote spinal segment.

## Case Presentation

### History

The patient in question was a 45-year-old Hispanic female who had lived in Canada for the previous 11 years. She reported a long history of manual labour and subsisted on similar occupations since arriving in Canada. At the time of her presentation, her occupation required prolonged periods of standing. The patient's chief complaint was chronic low back pain. There was no antecedent trauma, bone surgery, or history of cancer. The onset of low back pain was described as insidious, with constant achy pain of at least two years duration which was progressively worsening. The pain remained localised to the central lower back in the area of the lumbosacral junction. The pain was rated at 3/10 (verbal scoring) at its best and 8/10 (verbal scoring) at its worst. An increase in pain was associated with an increased level of physical activity during the day, with the pain typically worse in the evening. During periods of increased pain, there were intermittent incidences of pain radiating to the right-sided posterior thigh and leg with "pins and needles" in the lateral toes of the right foot. Treatment to date had consisted of non-steroidal anti-inflammatory medication, which she felt had been of limited benefit. There had been no use of acupuncture, massage therapy, therapeutic exercise, or any manual therapies for this condition. The patient was referred to the department of Medical Imaging for evaluation of chronic lower back pain.

The patient reported that no spinal imaging had been performed in investigation of these complaints. This was confirmed by a review of the patient's records.

### Physical Examination

Imaging specialists typically do not examine patients who are referred into an imaging department for investigation. As such, no physical examination was performed in this case. Permission was granted after the images were interpreted only to interview the patient for this case report.

### Imaging Protocol

A three-phase bone scan was performed with ^99 m^Tc(Technetium)-MDP (methylene diphosphonate) including blood flow and blood pool imaging followed by a delayed whole-body scan. SPECT/CT imaging centered over the lumbar spine was subsequently performed on a Symbia T6 (Siemens), a dual-head gamma-camera incorporating a low-dose 6-slice non-contrast enhanced CT (12 mAs, 130 kVp, Effective Dose < 4 mSv). The CT scan duration was less than 1 min. Overall, the SPECT/CT scan duration was about 20 min. The SPECT/CT fused images were displayed on the e-soft 2007 workstation (Siemens) in axial, sagittal, and coronal slices.

### Imaging Findings

**Figure 1:**
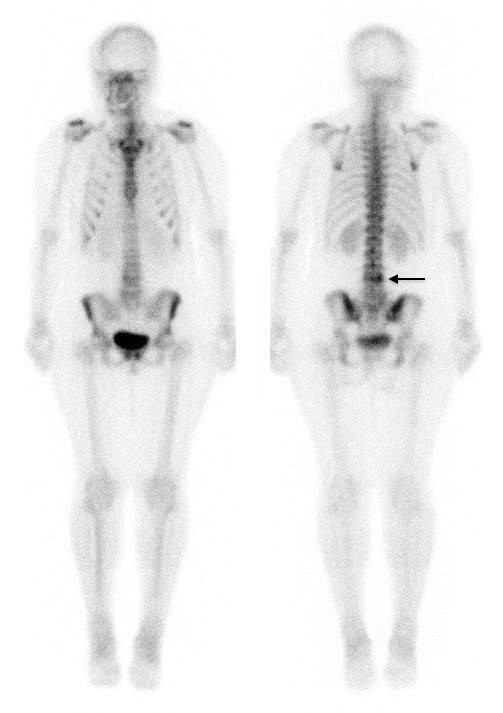
Delayed Whole Body images, anterior (L) and posterior showing focal tracer uptake in right L3/L4 facet joint region (continuous black arrow).

The blood flow and pool images were unremarkable, suggesting no active inflammatory process. The delayed whole-body images showed degenerative changes in multiple sites in the axial and appendicular skeleton. Heterogeneous tracer uptake was noted at multiple spinal levels with marked increased focal tracer uptake in the right lateral aspect of L3 (Figure 1).

**Figure 2:**
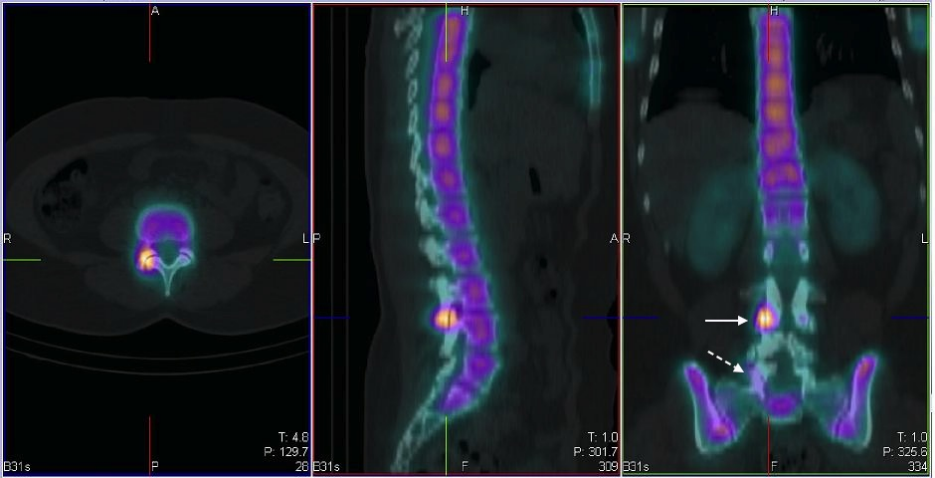
**SPECT/CT images (axial, sagittal, coronal) localizing intense, focal tracer uptake to the right L3/L4 facet joint (continuous arrow)**. Note is made of mild tracer uptake in the right L5/S1 facet joint (dashed arrow).

**Figure 3:**
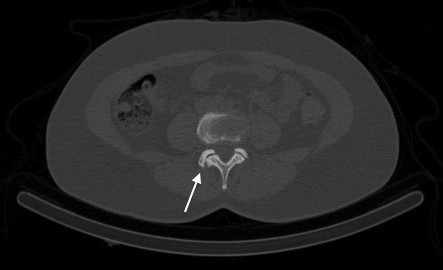
CT image from SPECT/CT demonstrating mild degenerative changes at right L3/L4 facet joint (white arrow).

**Figure 4:**
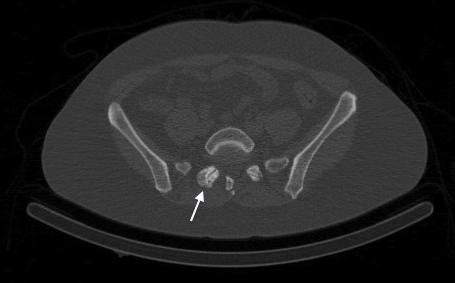
CT image from SPECT/CT demonstrating marked degenerative arthrosis at right L5/S1 facet (white arrow).

**Figure 5:**
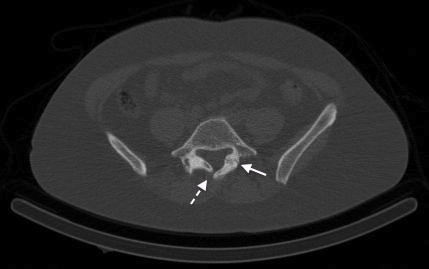
CT image from SPECT/CT demonstrating discontinuity of the left pars interarticularis (continuous arrow) and an incidental spina bifida occulta (dashed arrow).

SPECT/CT images confirmed intense tracer uptake in the right L3/L4 facet joint (Figure 2). In the absence of other osteoblastic or osteolytic pathology, this is most consistent with active degenerative osteoarthrosis. Mild tracer uptake is also noted in the right facet joint of L5/S1 in the presence of marked degenerative arthrosis, which is consistent with limited active bony pathology. The low-dose CT images from the SPECT/CT are not of diagnostic quality but they are adequate for anatomical localisation and gross tissue evaluation. In reviewing the axial CT images, there are many abnormalities to note. At the level of the L3/L4 right facet joint, there are only mild indicators of degenerative joint disease: focal joint space narrowing and early sclerosis (Figure 3). In addition to marked degeneration of the right L5/S1 facet (Figure 4), there is a left-sided discontinuity of the pars interarticularis and an incidental spina bifida occulta at L5 (Figure 5).

## Discussion

As a category, mechanical low back pain accounts for up to 97% of low back pain diagnoses [1,2]. A diagnosis of mechanical low back pain implies that there are no vascular, infectious, inflammatory or neoplastic etiologies underlying the patient's complaints but does little to clinically isolate a specific source of pain or identify a definitive avenue of treatment. This diagnosis encompasses a broad subset of possible tissue pathologies, many of which cannot be accurately diagnosed by physical examination. This may limit a manual therapist's ability to specifically prescribe a treatment regimen or accurately predict a response to treatment.

Lumbar facet joint capsules are richly innervated with nociceptive and autonomic nerve fibers and, as such, are a potential source of low back pain [3]. Despite a broad range of reported prevalence, this position is generally accepted and is supported by investigations that have injected facet joints with corticosteroids and anesthetic agents and demonstrated success in relieving some low back pain [4].

Undifferentiated lower back pain is a well established clinical indication for planar/SPECT bone scintigraphy [5,6]. Integrated SPECT/CT imaging is useful for anatomic and functional evaluation of benign and malignant spine bone diseases, particularly for evaluation of chronic low back pain [7-13]. The use of a low radiation dose multislice CT for 3D anatomic localisation of disk and facet degenerative disease improves the diagnostic accuracy and the specificity of planar/SPECT bone scintigraphy [11-13]. The CT images will also provide additional 3D detail about anatomic structures included in the region of interest that do not actively uptake radiotracer. The CT component of SPECT/CT provides a lower radiation dose than diagnostic multi-detector CT imaging, with an effective radiation dose of less than 4 mSv generating a radiation burden in the order of the yearly natural background exposure (approximately 3 mSv). The fast CT scanner (less than 1 min) may be used routinely for anatomic mapping in bone scintigraphy procedures [10].

This case highlights several interesting findings for the clinical setting and raises a number of potential research opportunities. Perhaps the most impressive finding is the demonstration of metabolically active bone in a facet joint with minimal overt degenerative changes that likely would not have been identified as pathological on plain radiographs. While it is well documented that clinical symptoms do not correlate well with radiographic and multidetector CT findings [2,14,15], the increased radiotracer uptake found on the SPECT part from SPECT/CT may be due to painful facet arthropathy, ongoing degenerative changes or the chronic sequelae of adverse mechanical loading. This case has also demonstrated that facet hypertrophy found on CT does not correlate with SPECT positivity, suggesting that facet hypertrophy has either a latent period or represents an end-point as part of the degenerative process. It is possible that by the time facet hypertrophy is notable on anatomic imaging, the metabolic activity of the bone is normalising, as demonstrated by the limited tracer uptake at the right L5/S1 facet joint. Rehabilitative treatment directed at SPECT positivity may also allow potentially corrective conservative intervention before advanced degenerative changes occur, theoretically reducing the likelihood of disability due to profound alteration of joint mechanics.

In this particular patient's clinical presentation, the CT findings of L5/S1 facet joint hypertrophy coupled with paresthesias to the right posterior thigh and leg and the lateral toes of the right foot is suggestive of a localised lesion to the L5/S1 segment, possibly affecting the right S1 nerve root. Because the SPECT findings do not reveal any significant active bony uptake in this region, any pain arising from this level may be due to non-osseous tissues being irritated, such as pain sensitive soft tissues (disc, ligament, muscle) or the exiting nerve root being affected by discal pathology and the hypertrophied facet demonstrated at this level. CT provides limited resolution of soft tissues in spine imaging and a more thorough evaluation of soft tissues would require the superior tissue resolution of MRI. However, given the findings of intense tracer uptake at the L3/L4 facet, the clinical presentation of low back pain may be due to local facet joint pathology at L3/L4 or a combination of pathologies in different tissues at multiple levels.

For manual therapists, one possible algorithm of investigation and treatment in such cases is to coordinate physical assessment with diagnostic or therapeutic facet joint injections for metabolically active facet joints found by SPECT/CT [15]. This approach can be beneficial by localizing the source of low back pain if the injection relieves the chief complaint. This approach may also be used in conjunction with manual therapies and targeted rehabilitation to improve the biomechanical function of the spine through muscle strengthening and muscle recruitment, and to improve joint mobility via mobilization and/or manipulation while the patient is experiencing pain relief from a therapeutic injection [2].

With the ability of SPECT/CT to identify sites of active bony metabolism, a role in manual therapy research becomes evident. The ability to objectively document sites of active biomechanical stress or degeneration opens up the possibility of using SPECT/CT to assess the effectiveness of manipulative and stabilization therapies. If such therapies are able to affect the biomechanics and stability of the spine, then SPECT/CT offers an avenue for objective assessment of their effectiveness. While such research may be technically and ethically difficult, SPECT/CT documentation of facet arthropathy has the potential to support the hypothesis that improving biomechanical function may both relieve pain and affect the degenerative process.

## Conclusions

Facet arthropathy is a commonly accepted causative or contributing agent to low back pain syndromes. The ability of integrated SPECT/CT to precisely localise metabolically active facet joints may provide direction of treatment to manual therapies focused on improving spinal function. It is postulated here that improvements in biomechanical function, accompanied by patient subjective improvement, may demonstrate improvement or resolution of SPECT/CT findings of facet arthropathy. Research would have to be carefully designed to test this hypothesis.

## Consent

Written informed consent was obtained from the patient for publication of this case report and any accompanying images. A copy of the written consent has been provided to the Editor-in-Chief of this journal.

## Competing interests

The authors declare that they have no competing interests.

## Authors' contributions

MC performed the literature review, interviewed the patient and prepared the manuscript. JLU, MA and TB contributed to the drafting of the manuscript as well as critical review and image interpretation. All authors approved of the final manuscript
